# Four and a half Lin11, Isl-1, Mec-3(LIM) domain protein 1 (FHL1) regulates vascular remodeling in low-flow vein grafts

**DOI:** 10.7717/peerj.21104

**Published:** 2026-04-14

**Authors:** Chaoqun Wang, Jiantao Chen, Guangxian Chen, Jianbo Liao, Suiqing Huang, Kangni Feng, Ziyin Ye, Mengya Liang, Zhongkai Wu

**Affiliations:** 1 Department of Cardiac Surgery, First Affiliated Hospital of Sun Yat-Sen University, Guangzhou, China; 2 NHC Key Laboratory of Assisted Circulation, Sun Yat-Sen University, Guangzhou, China; 3 Department of Pathology, First Affiliated Hospital of Sun Yat-Sen University, Guangzhou, China

**Keywords:** FHL1, Neointimal hyperplasia, Low-flow, Vein graft remodeling

## Abstract

**Background:**

Neointimal hyperplasia (NIH) is a critical determinant of long-term patency of vein grafts following coronary artery bypass grafting (CABG). The development of neointima is influenced by hemodynamic conditions. Our previous studies have demonstrated that four and a half LIM domain protein 1 (FHL1) effectively attenuates neointimal formation in vein grafts under normal flow conditions. However, the role of FHL1 in low-flow hemodynamic environments remains unclear. Elucidating the mechanisms underlying NIH in low-flow settings is essential for improving low-flow vein graft outcomes.

**Methods:**

The vein graft model was established in Sprague-Dawley (SD) rats, with low blood flow induced by ligation of the internal carotid artery. Then eointimal area was quantified using histological staining, while the expression of neointimal markers was assessed *via* immunofluorescence analysis. FHL1-knockout SD rats and SD rats with adenovirus-mediated FHL1 overexpression were used to evaluate the progression of NIH under low-flow conditions.

**Results:**

Following internal carotid artery ligation, the mean blood flow velocity in the vein grafts decreased immediately (*p* < 0.0001), resulting in a significant increase in the neointimal area at 28 days post-surgery (*p* < 0.05). In FHL1-knockout rats under low-flow conditions, NIH was further exacerbated (*p* < 0.001), accompanied by marked downregulation of mature smooth muscle cell markers (*p* < 0.05) and decreased proliferative activity (*p* < 0.0001), reduced inflammatory mediators (*p* < 0.0001), and activation of the mitogen-activated protein kinase (MAPK) signaling pathway (*p* < 0.01). Conversely, FHL1 overexpression in low-flow grafts led to a reduction in neointimal thickening (*p* < 0.05), promoted phenotypic maturation of neointimal cells (*p* < 0.05), and suppressed proliferative activity (*p* < 0.05), MAPK pathway activation (*p* < 0.01), and inflammatory responses (*p* < 0.01).

**Conclusion:**

In low-flow vein grafts, FHL1 may promote favorable vascular remodeling by inhibiting the MAPK signaling pathway, thereby attenuating neointimal cell phenotype switching and mitigating vascular inflammation.

## Introduction

Obstructive arterial disease remains a major cause of global morbidity and mortality (Bottardi et al., 2024). In addition to balloon angioplasty, bypass grafting is a widely employed revascularization strategy for the treatment of obstructive arterial disease (Tang et al., 2022; Heng et al., 2023). Coronary artery bypass grafting (CABG) is currently the preferred revascularization approach for patients with left main coronary artery disease or severe three-vessel coronary artery disease (Amponsah & Fearon, 2025). Although recent advancements have reduced in postoperative mortality and CABG is now regarded as a safe and effective alternative to percutaneous coronary intervention (PCI), several challenges persist in optimizing surgical outcomes and long-term prognosis. Notably, venous graft restenosis is a major clinical challenge in the surgical management of coronary artery disease (Xenogiannis et al., 2021). Despite higher long-term patency rates associated with arterial grafts compared to saphenous vein grafts, the adoption of total arterial revascularization remains limited in clinical practice (Taggart et al., 2016). Consequently, the saphenous vein continues to be the most frequently utilized conduit in CABG procedures (Ghandakly, Tipton & Bakaeen, 2023; Zhou et al., 2024). The incidence of vein graft failure remains substantial, with approximately 50–60% of grafts developing restenosis or occlusion within 10 years post-surgery (Sabik 3rd, 2011).

Neointima formation in vein grafts was first described in 1965, and its development is known to be influenced by the hemodynamic environment (Glagov et al., 1995). Biomechanical forces within the vascular lumen, such as wall tension and shear stress, which are generated by blood flow velocity, have been established as critical regulators of vascular remodeling and intimal hyperplasia (Galt et al., 1993). Variations in surgical technique may reduce blood flow velocity, and the resulting low-flow hemodynamic milieu promotes neointimal hyperplasia. However, the underlying molecular mechanisms remain poorly understood, and no effective clinical interventions are currently available (Mattsson et al., 1997). Our previous studies have demonstrated that four and a half LIM domain protein 1 (FHL1) is stably and specifically expressed in the neointima and significantly attenuates neointima formation in vein grafts under normal blood flow conditions (Wang et al., 2024). FHL1 is characterized by the presence of four and a half LIM domains and is the founding member of the FHL family, which includes FHL1, FHL2, FHL3, FHL4, and FHL5 (Shathasivam, Kislinger & Gramolini, 2010; Lin et al., 2023; Qi et al., 2024).

In this study, a rat model of low-flow venous bypass grafts was employed to investigate the initiation and progression of neointimal hyperplasia under low-flow conditions. Furthermore, the functional role of FHL1 in neointima formation was systematically examined using both FHL1 knockout rats and local viral-mediated overexpression of FHL1, with the aim of expanding its potential therapeutic utility in vein graft failure.

## Materials and Methods

### Normal flow and low flow vein graft rat model

This study was approved by the Institutional Animal Care and Use Committee of Sun Yat-sen University and was conducted in accordance with the Guide for the Care and Use of Laboratory Animals (Approval No. SYSU-IACUC-2020-000074). The animals were housed in cages under controlled environmental conditions, including a stable temperature of 22 ± 2 °C, relative humidity of 45 ± 5%, and a 12-hour light-dark cycle. Standard laboratory rodent diet and water were provided ad libitum throughout the study.

**Figure 1:**
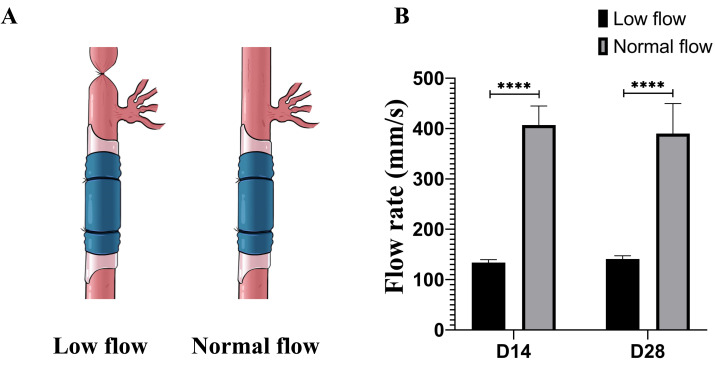
Schematic representation of vein bypass graft models under different flow conditions and corresponding blood flow measurements. (A) Schematic illustration of the surgical model for generating differential flow environments. The internal carotid artery branch was ligated distal to the common carotid artery, while the external carotid artery branch was preserved, thereby establishing a low-flow hemodynamic condition. (B) Quantitative comparison of blood flow volumes in the vein bypass grafts under different flow conditions following ligation of the internal carotid artery. ^∗∗∗∗^*p* < 0.0001.

As previously described (Wang et al., 2024), Sprague-Dawley (SD) rats weighing 250–350 g were obtained from Sun Yat-sen University (Guangzhou, Guangdong, China). Pentobarbital (Sanofi, Paris, France) was administered at a dose of 25 mg/kg to induce anesthesia. Full systemic anticoagulation was achieved by intravenous injection of 100 units of heparin. Under a microscope (DWYQ-012; KERONG, Huizhou, China) and sterile conditions, a segment of the right external jugular vein was surgically removed and immediately placed in heparinized saline (5000 U/L) for preservation. The right common carotid artery was then exposed and clamped at both ends. A cuff technique using plastic tubes (Smiths Medical, Birmingham, UK) was used to create the vein graft model. The arterial end was everted over the cuff and tied with an 8-0 silk suture. Subsequently, the inverted vein was centered in the artery and secured with another silk ligature. To create a low-flow vein graft, the internal carotid artery was ligated while preserving the four external carotid branches (Fig. 1A). Vein grafts in the designated group were locally incubated with poloxamer gel containing Ad-FHL1 (HBAD-Adeasy-h-FHL1-3xflag-EGFP; 7 × 10^10^ PFU/ml), while controls received Ad-NC (HBAD-EGFP). These were synthesized by HanBio (Shanghai, China). The FHL1 sequence inserted into the adenovirus was previously described (Wang et al., 2024) (see Sequence File). Surgical incisions were closed with 6-0 sutures. Graft blood flow was measured using an ultrasonic flowmeter. Aspirin (MFCD00002430; Merck, Rahway, NJ, USA) was given orally at 20 mg/day to prevent thrombosis and to implement postoperative analgesia. Penicillin (A6920-5g; Solarbio, Beijing, China) was administered for three days post-surgery to prevent infection. All rats were euthanized by overdose of pentobarbital (300 mg/kg; Sanofi, Paris, France). The criteria for euthanasia ensured that all animals were humanely euthanized and death was confirmed immediately. All animals met these criteria. Patent grafts from each group were harvested at each time point for morphological analysis and fixed in 10% formalin.

The rats that did not undergo any surgical procedure were named as “Ctrl” (*n* = 5) group. The rats that did not undergo ligation of the internal carotid arteries were named as “Normal flow FHL(+)” (*n* = 5) group. The rats that underwent ligation of the internal carotid arteries were named as the “Low flow FHL(+)” (*n* = 5) group. The rats that underwent ligation of the internal carotid arteries and were incubated with poloxamer gel containing Ad-FHL1 were named as the “Low flow Ad-FHL” (*n* = 5) group. The rats that underwent ligation of the internal carotid arteries and were incubated with poloxamer gel containing Ad-NC were named as the “Low flow Ad-NC” (*n* = 5) group. The experimental unit was the individual rat. No unexpected complications or perioperative mortality were observed.

### Creation of FHL1 gene-knockout rats

As previously described (Wang et al., 2024), wild-type (WT) controls were established using normal Sprague-Dawley (SD) rats. The FHL1 gene (Gene ID: 25177) is located on the rat X chromosome. The genomic region spanning exons 3–7 was chosen as the target site. FHL1 KO rats were generated using CRISPR/Cas9 technology as follows: zygotes at the one-cell stage were collected. Cas9 mRNA and guide RNA (gRNA) were produced by *in vitro* transcription using commercial kits (AM1354+AM1908; Thermo Fisher Scientific, Waltham, MA, USA). A microinjection mix containing 0.3 µM Cas9 mRNA and 0.75 µM gRNA was delivered into fertilized eggs. These cells were cultured in KSOM medium (Millipore, Germany), and the two-cell stage cells were surgically transferred into the recipient females (Charles River Laboratories). F0 founders were confirmed by PCR, then mated with WT rats to produce F1 offspring. F2 homozygotes were obtained from F1 heterozygotes. Genomic DNA was extracted from tail samples, and the PCR product length for the FHL1 knockout rats was 488 bp, compared with 778 bp in wild-type rats. The gRNA sequences and genotyping primers used were as previously described (Wang et al., 2024) (see Sequence File). FHL1 KO rats that underwent ligation of the internal carotid arteries were designated as the “Low flow FHL(-)” group (*n* = 3).

### Morphometric assessment

External jugular veins were harvested as controls. Sections (3 µm thick) were collected from a region 0.5 mm distal to the proximal cuff anastomosis and processed for paraffin embedding. Morphological evaluation was performed using hematoxylin and eosin (H&E) staining (G1005-500; Servicebio Technology, Wuhan, China) and Masson’s trichrome staining (G1006-100; Servicebio Technology, Wuhan, China). The samples were stained with H&E or Masson solution according to the manufacturer’s protocol. The luminal area was defined as the space bounded by the internal elastic lamina and the luminal surface. The neointimal area was calculated as the difference between these two measurements.

### Immunofluorescence and immunohistochemistry staining

Paraffin-embedded vascular tissue sections were deparaffinized and rehydrated through a standard series of solvents. Antigen retrieval was performed using citrate buffer (pH 6.0) with microwave assistance. Sections were then blocked with 5% donkey serum at 37 °C for 30 min to reduce non-specific binding. Primary antibodies (Table 1) were applied and incubated overnight at 4 °C. After thorough PBS washes, the tissues were incubated at room temperature with secondary antibodies and DAPI (G1407-25; Servicebio Technology) under gentle agitation. Following rinsing, the sections were cover slipped using Fluoromount-G mounting medium (0100-20; Southern Biotech). Immunofluorescence and immunohistochemistry images were analyzed using the ImageJ software (National Institutes of Health, Bethesda, MD, USA). Quantification was based on immunofluorescence and immunohistochemistry intensity normalized to the tissue area, and statistical graphs represent the fluorescence and histochemistry signals per unit area.

### Statistical analysis

All statistical evaluations were performed using GraphPad Prism 8.0 (GraphPad Software, Inc., San Diego, CA, USA), and the analysts were blinded to the group assignments. The normality of the data distribution was determined using the Pearson normality test (*α* = 0.05). For data following a normal distribution, comparisons between the two groups were made using a two-tailed Student’s *t*-test; for non-normal distributions, the Mann–Whitney *U* test was applied. When comparing multiple groups, one-way analysis of variance (ANOVA) was used for normally distributed data, and the Kruskal–Wallis test was used for non-normally distributed data. *Post hoc* analysis following ANOVA was conducted using the Bonferroni correction. Data are expressed as the mean ± standard deviation. A *p*-value less than 0.05 was considered statistically significant.

## Results

### Hemodynamic parameters of the low-flow rat model and neointimal changes following FHL1 knockout

**Figure 2:**
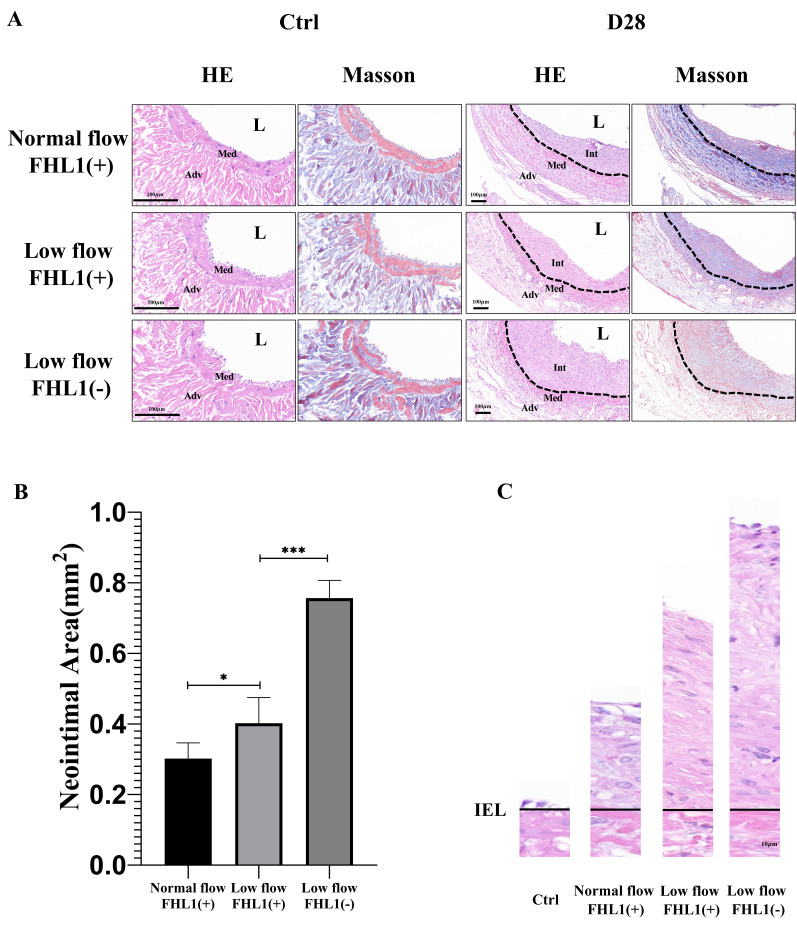
Histopathological and neointimal area analysis of vein grafts under normal flow, low flow and FHL1-deficient low flow conditions. (A) Hematoxylin and eosin (H&E) and Masson’s trichrome staining of vein grafts at 28 days post-surgery in the normal flow (*n* = 5), low flow (*n* = 5), and FHL1-deficient low flow (*n* = 3) groups. Int, neointima of the vein graft; Med, media tunica of the vein graft; Adv, adventitia of the vein graft; L, lumen. Scale bar = 100 µm. (B) Bar graph depicting neointimal area in vein grafts across the three experimental groups at 28 days post-surgery. (C) Representative H&E-stained cross-sections of vein grafts demonstrating progressive neointimal formation in each group. The solid line delineates the internal elastic lamina. ^∗^*p* < 0.05 and ^∗∗∗^*p* < 0.001.

After successful establishment of the low-flow vein graft model, blood flow was reduced to approximately one-third of that in the normal-flow model. The hemodynamic conditions in this model remained stable and were sustained for up to 28 days postoperatively (Fig. 1B, *p* < 0.0001). At 28 days after surgery, the neointimal area in low-flow vein grafts was significantly greater than that in normal-flow grafts (*p* < 0.05). Moreover, FHL1 knockout further exacerbated neointima formation compared with WT controls under low-flow conditions (*p* < 0.001) (Figs. 2A–2B). To enable direct comparison, the internal elastic laminae were aligned across sections, and neointimal development in the three experimental groups was visualized using H&E staining (Fig. 2C).

**Table 1:** Antibodies used for immunofluorescence and immunohistochemistry staining.

Antibody	Cat#	Manufacturer	Dilution
anti-*α*-SMA	ab124964	Abcam, Cambridge, UK	1:250
anti-FHL1	ab255828	Abcam, Cambridge, UK	1:50
anti-PCNA	ab92552	Abcam, Cambridge, UK	1:30
anti-Vimentin	GB12192	Servicebio Technology, Wuhan, China	1:200
anti-CD68	ab283654	Abcam, Cambridge, UK	1:100
anti-VCAM-1	ab134047	Abcam, Cambridge, UK	1:250
anti-Cnn1	ab46794	Abcam, Cambridge, UK	1:300
anti-MAPK	ab170099	Abcam, Cambridge, UK	1:200
anti-SMMHC	GB11805	Servicebio Technology, Wuhan, China	1:250

### Under low-flow conditions, FHL1 deficiency exacerbated neointimal cell activation and promoted inflammatory responses

**Figure 3:**
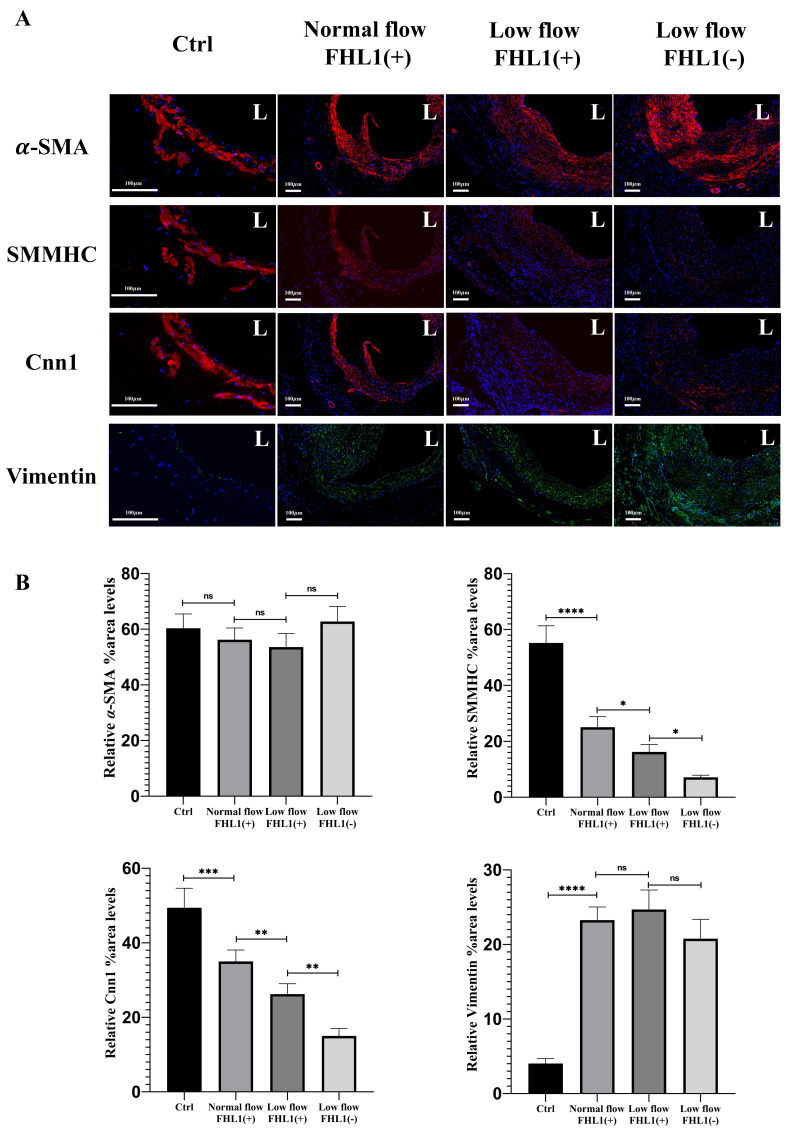
Immunofluorescence characterization and quantitative analysis of neointimal cell markers (*α*-SMA, SMMHC, Cnn1 and Vimentin) in vein grafts under normal flow, low flow and FHL1-deficient low flow conditions. (A) Immunofluorescence (IF) images of DAPI (blue), *α*-smooth muscle actin (*α*-SMA; red), smooth muscle myosin heavy chain (SMMHC; red), Calponin-1 (Cnn1; red) and Vimentin (green) staining in vein grafts at 28 days post-surgery under normal flow, low flow, and FHL1-deficient low flow conditions. L, lumen. Scale bar = 100 µm. (B) Quantitative analysis of the average regional expression intensity of neointimal hyperplasia (NIH) for *α*-SMA, SMMHC, Cnn1 and Vimentin under normal flow (*n* = 5), low flow (*n* = 5), and FHL1-deficient low flow (*n* = 3) conditions. ns, no significance, ^∗^*p* < 0.05, ^∗∗^*p* < 0.01, ^∗∗∗^*p* < 0.001, and ^∗∗∗∗^*p* < 0.0001.

**Figure 4:**
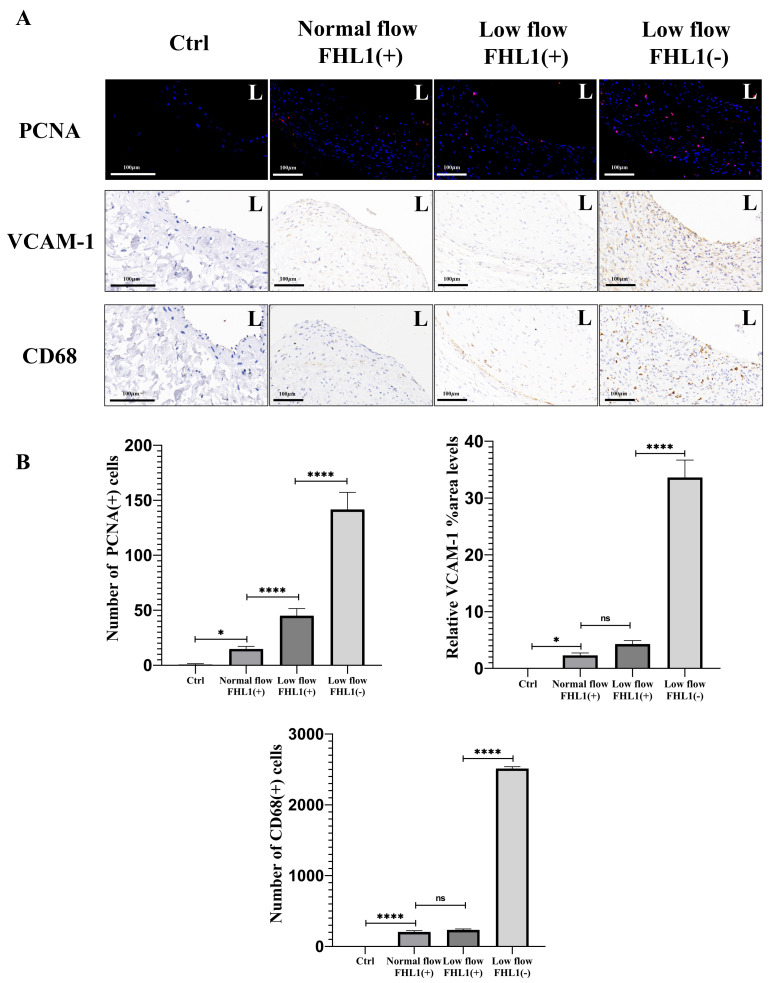
Immunofluorescence and immunohistochemical characterization and quantitative analysis of neointimal cell markers (PCNA, VCAM-1 and CD68) in vein grafts under normal flow, low flow and FHL1-deficient low flow conditions. (A) IF images of DAPI (blue) and proliferating cell nuclear antigen (PCNA; red) staining and Immunohistochemical (IHC) images of vascular cell adhesion molecule-1 (VCAM-1) and macrophage marker (CD68) expression in vein grafts at 28 days post-surgery across all three groups. (B) Quantitative analysis of the average regional expression intensity of neointimal hyperplasia (NIH) for VCAM-1 and number of PCNA(+), CD68(+) cells under normal flow (*n* = 5), low flow (*n* = 5), and FHL1-deficient low flow (*n* = 3) conditions. ns, no significance, ^∗^*p* < 0.05, ^∗∗^*p* < 0.01, ^∗∗∗^*p* < 0.001, and ^∗∗∗∗^*p* < 0.0001.

Compared with normal-flow conditions, *α*-SMA and vimentin expression showed no significant differences; however, the expression of myogenic differentiation markers SMMHC (*p* < 0.05) and Cnn1 (*p* < 0.01) was reduced, accompanied by enhanced proliferative activity (*p* < 0.0001). In low-flow vein grafts with FHL1 knockout, the downregulation of SMMHC (*p* < 0.05) and Cnn1 (*p* < 0.01) was further accentuated, and cellular proliferation was markedly increased (*p* < 0.0001) (Figs. 3 and 4). In neointimal cells of low-flow vein grafts, a mild upregulation of the inflammatory adhesion molecule VCAM-1 and minimal macrophage infiltration were observed. Notably, FHL1 deficiency significantly amplified the expression of inflammatory adhesion molecules (*p* < 0.0001) and induced pronounced macrophage infiltration (*p* < 0.0001) (Fig. 4). These findings demonstrate that under low-flow hemodynamic conditions, loss of FHL1 enhances the transition of neointimal cells toward a myofibroblast-like phenotype, increases the expression of pro-inflammatory adhesion molecules, and exacerbates macrophage recruitment in vein grafts.

### Under low-flow conditions, FHL1 modulated the expression of downstream signaling pathways

**Figure 5:**
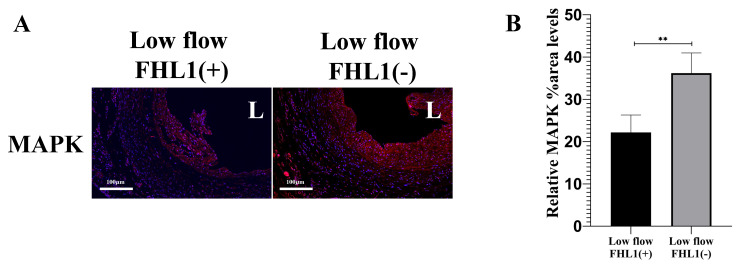
Differential pathway activation in vein grafts under FHL1 related low-flow conditions. (A) IF images and (B) quantitative analysis of DAPI (blue) and mitogen-activated protein kinase (MAPK; red) staining in vein grafts at 28 days post-surgery under low flow (*n* = 5) and FHL1-deficient low flow (*n* = 3) conditions. L, lumen. Scale bar = 100 µm. ^∗∗^*p* < 0.01.

Previous spatial transcriptomic analyses revealed that neointima development may be associated with the activation of several key signaling pathways, including the mitogen-activated protein kinase (MAPK), PI3K–Akt, and Wnt pathways (Wang et al., 2024). The MAPK signaling pathway regulates a wide range of cellular processes. Previous evidence suggests a regulatory interaction between FHL1 and the MAPK signaling pathway (Li et al., 2008). Notably, under low-flow hemodynamic conditions, FHL1 deficiency resulted in enhanced activation of the MAPK signaling pathway, as indicated by increased expression of pathway-related protein (*p* < 0.01; Fig. 5). These findings support a regulatory mechanism between FHL1 and the MAPK signaling pathway in low-flow vein graft remodeling.

### Adenovirus-mediated FHL1 overexpression attenuates neointimal hyperplasia in low-flow vein grafts

**Figure 6:**
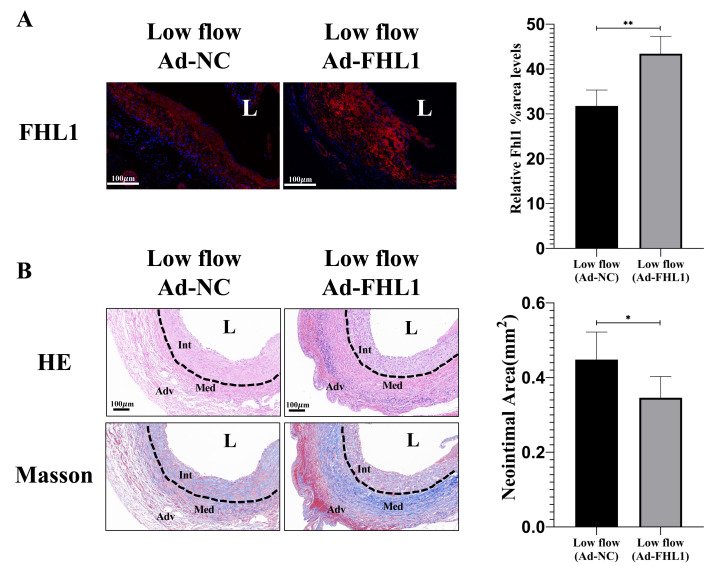
FHL1 overexpression attenuates neointimal hyperplasia (NIH) under low-flow hemodynamic conditions. (A) Immunofluorescence (IF) detection of FHL1 expression and quantitative analysis of FHL1 staining intensity in the Ad-NC and Ad-FHL1 groups at 28 days post-surgery under low-flow conditions (*n* = 5 per group). (B) H&E and Masson staining and comparative assessment of neointimal area in the Ad-NC and Ad-FHL1 groups at 28 days post-surgery under low-flow conditions. ^∗^*p* < 0.05, ^∗∗^*p* < 0.01.

**Figure 7:**
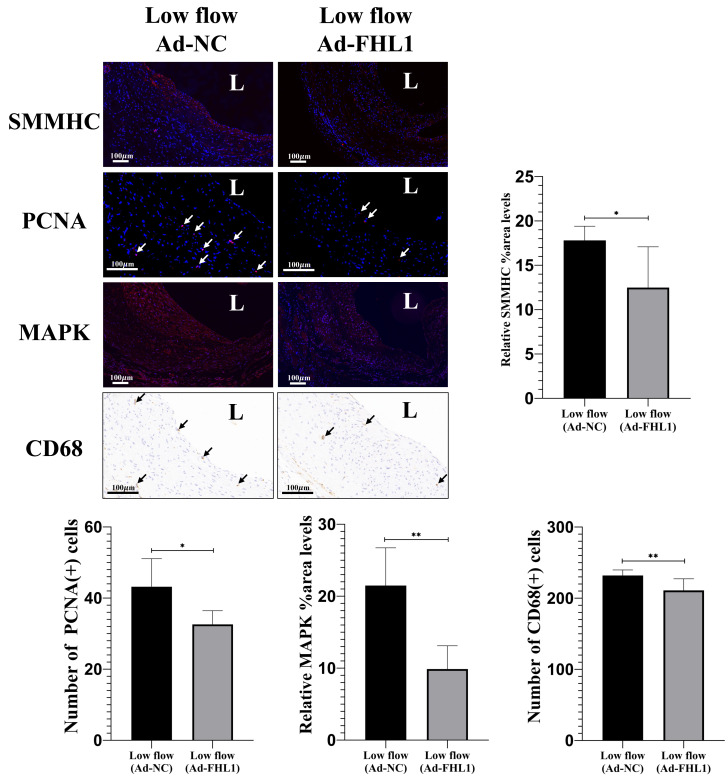
FHL1 overexpression causes neointimal cell markers changes under low-flow hemodynamic conditions. IF and IHC images and quantitative analysis of smooth muscle myosin heavy chain (SMMHC; red), proliferating cell nuclear antigen (PCNA; red), mitogen-activated protein kinase (MAPK; red), and macrophage marker (CD68) expression in the Ad-NC and Ad-FHL1 groups at 28 days post-surgery under low-flow conditions. L, lumen. Scale bar = 100 µm. ^∗^*p* < 0.05, ^∗∗^*p* < 0.01.

Compared with Ad-NC, Ad-FHL1 markedly increased FHL1 expression in the neointima of low-flow vein grafts, confirming efficient viral transduction (Fig. 6A, *p* < 0.01). Subsequently, neointimal area and marker expression were assessed 28 days post-surgery in grafts with FHL1 overexpression. FHL1 overexpression significantly reduced neointima under low-flow hemodynamic conditions (Fig. 6B, *p* < 0.05). Furthermore, it promoted the expression of mature smooth muscle cell markers (*p* < 0.05) and downregulated the proliferative marker PCNA (*p* < 0.05; Fig. 7). In addition, FHL1 overexpression reduced macrophage recruitment (*p* < 0.01), and inhibited activation of the MAPK signaling pathway (*p* < 0.01; Fig. 7). Collectively, these findings demonstrated that, under low-flow hemodynamic conditions, FHL1 attenuated neointimal hyperplasia by inhibiting the MAPK signaling pathway, suppressing neointimal cell activation, and mitigating inflammatory responses.

## Discussion

Restenosis of venous bypass grafts is a multifactorial pathological process in which neointimal hyperplasia is the primary determinant of long-term saphenous vein graft patency (Yu et al., 2025; Tierney et al., 2021; Zhan et al., 2024). Vein grafts exhibit dynamic responses to hemodynamic cues after grafting (Galt et al., 1993). Vein graft failure significantly increases the risk of adverse cardiovascular events, posing a serious threat to patient health and imposing substantial socioeconomic and familial burdens (Zhong et al., 2021). In clinical practice, inter-operator variability in surgical techniques may result in suboptimal anastomoses, leading to low blood flow in certain vein grafts. This study aimed to investigate whether targeted genetic intervention can reduce the long-term occlusion rate of low-flow vein grafts without the need for secondary surgical revision. Currently, there remains a critical gap in understanding the pathophysiological mechanisms underlying low-flow venous graft failure (Mattsson et al., 1997). Therefore, an in-depth investigation of strategies for mitigating occlusion in low-flow vein grafts holds significant clinical and translational importance.

*In vitro* cell and *ex vivo* venous tissue culture systems are unable to adequately recapitulate the complex interplay of factors involved in *in vivo* remodeling. Consequently, animal models remain essential tools for investigating the pathogenesis of vein graft restenosis (Swartz & Andreadis, 2013). By ligating the internal carotid artery branch distal to the common carotid artery, a stable and reproducible low-flow hemodynamic environment is created at the anastomotic site. After implantation, the transplanted vein undergoes adaptive remodeling under arterial hemodynamic forces at varying flow rates, providing a robust experimental platform for studying venous grafts under different flow conditions. Evidence indicates that when veins are grafted into the venous system, neointimal formation does not occur, highlighting the critical role of the hemodynamic environment in driving neointima development. Under low-flow conditions, venous grafts exhibit a significantly increased neointimal area (Sterpetti et al., 1998). Our previous studies have demonstrated that FHL1 effectively attenuates neointimal hyperplasia under normal flow conditions (Wang et al., 2024). Therefore, this study aimed to investigate whether FHL1 retains its regulatory influence on neointimal formation under low-flow hemodynamic conditions.

Immunohistochemistry and immunofluorescence offer distinct advantages in visualizing marker expression within the spatial architecture of the neointima. Staining results revealed that neointimal cells under normal flow conditions exhibited characteristic myofibroblast phenotypes, marked by reduced expression of mature smooth muscle markers and increased expression of proliferative markers. Under low-flow conditions, these myofibroblast features were further enhanced; notably, in the context of both low flow and complete FHL1 deficiency, activation of the myofibroblast phenotype in neointimal cells was significantly amplified. These findings are consistent with previous studies demonstrating that during tissue repair, a subset of cells is activated and differentiates into myofibroblasts, exhibiting a hybrid phenotype with both synthetic and contractile properties (Jolly et al., 2022). Myofibroblasts are highly contractile cells whose phenotype lies between fibroblasts and vascular smooth muscle cells, and they display pro-inflammatory and proliferative activities (Otani et al., 2000). However, in the setting of venous remodeling, a shift toward a pro-inflammatory and hyperproliferative state is detrimental, because exacerbated inflammation and excessive cell proliferation contribute to luminal narrowing, a hallmark of pathological vascular remodeling (Forte et al., 2010).

FHL1 is characterized by a tandem array of four complete LIM domains and an N-terminal single zinc finger domain that shares sequence homology with the C-terminal region of the LIM domain motif (Shathasivam, Kislinger & Gramolini, 2010). Its cellular functions appear to be both cell type–and tissue-specific. At the subcellular level, FHL1 was initially identified as being predominantly localized in the cytoplasm, where it associates with focal adhesions and participates in biomechanical stress responses (Cao et al., 2022); it is therefore considered a scaffold component of the mechanosensory complex (Wang et al., 2018). The inhibitory effects of FHL1 on cell growth and migration have been demonstrated in multiple tumor cell lines, supporting its role as a tumor suppressor (Tao et al., 2024; Zeng & Jiang, 2024). The MAPK signaling pathway has been implicated in neointimal formation, where it regulates vascular cell phenotypic modulation, contraction, and proliferation (Xiang et al., 2023). In addition, macrophages play critical roles in tissue injury and repair. As key mediators of innate immunity, they contribute to adaptive immune regulation and are widely distributed throughout various tissues and organs (Liu et al., 2020). Their primary functions include phagocytosis, clearance of cellular debris and pathogens, and modulation of immune responses (Wang et al., 2007). Macrophages display remarkable morphological and functional diversity and plasticity, enabling them to adapt their phenotype in response to cues such as cellular origin, local tissue microenvironment, and external stimuli (Wolff et al., 2004).

This study conducted the first investigation into the role of FHL1 in neointima formation in low-flow venous grafts, employing both FHL1 knockout rats and viral-mediated overexpression of FHL1 in vein grafts. In FHL1-deficient low-flow vein grafts, the MAPK signaling pathway was markedly upregulated, promoting neointimal cell activation toward a myofibroblast phenotype, enhancing the expression of inflammatory adhesion molecules and macrophage recruitment, and consequently increasing the neointimal area. In contrast, FHL1-overexpressing grafts exhibited opposing effects. These findings indicate that, in low-flow vein grafts, FHL1 exerts regulatory control over the MAPK signaling pathway, thereby modulating neointimal cell activation and vascular inflammatory responses, ultimately suppressing neointima formation under low-flow hemodynamic conditions. These results provide direct *in vivo* evidence for a protective role of FHL1 in low-flow vein graft remodeling, thereby expanding its potential therapeutic applications in vein graft failure.

## Limitation

This study focused on FHL1, but low-flow vascular remodeling involves multiple genes and pathways beyond FHL1. In addition, this study l did not include *in vitro* mechanistic experiments to delineate the direct molecular mechanisms of FHL1. To address this limitation, future studies should incorporate *in vitro* assays to further define how FHL1 regulates the low-flow vein graft remodeling.

## Conclusion

In summary, this study investigated how FHL1 modulates neointimal hyperplasia in low-flow vein grafts. We demonstrated that FHL1 is linked to the phenotypic transition of neointimal cells and activation of vascular inflammatory responses. These results suggest that FHL1 is a promising therapeutic target for mitigating low-flow–driven neointimal development.

##  Supplemental Information

10.7717/peerj.21104/supp-1 Supplemental Information 1 ARRIVE 2.0 checklist

10.7717/peerj.21104/supp-2 Supplemental Information 2 Sequences

## Additional Information and Declarations

### Data Availability

The following information was supplied regarding data availability: The data is available at figshare: Wang, Chaoqun (2025). Four and a half LIM domain protein 1(FHL1) regulates vascular remodeling in low-flow vein grafts. figshare. Dataset. https://doi.org/10.6084/m9.figshare.30646400.v2.
