# Supplementary material for: Four and a half Lin11, Isl-1, Mec-3(LIM) domain protein 1 (FHL1) regulates vascular remodeling in low-flow vein grafts
Source: PeerJ. 2026 Apr 14;14:e21104. doi: 10.7717/peerj.21104 (PMC13089220; doi:10.7717/peerj.21104)
Supplement: Supplemental Information 2 [file peerj-14-21104-s002.docx]

The sequence of FHL1 inserted was as follows:

atggcggagaagtttgactgccactactgcagggatcccttgcaggggaagaagtatgtgcaaaaggatggccaccactgctgcctgaaatgctttgacaagttctgtgccaacacctgtgtggaatgccgcaagcccatcggtgcggactccaaggaggtgcactataagaaccgcttctggcatgacacctgcttccgctgtgccaagtgccttcaccccttggccaatgagacctttgtggccaaggacaacaagatcctgtgcaacaagtgcaccactcgggaggactcccccaagtgcaaggggtgcttcaaggccattgtggcaggagatcaaaacgtggagtacaaggggaccgtctggcacaaagactgcttcacctgtagtaactgcaagcaagtcatcgggactggaagcttcttccctaaaggggaggacttctactgcgtgacttgccatgagaccaagtttgccaagcattgcgtgaagtgcaacaaggccatcacatctggaggaatcacttaccaggatcagccctggcatgccgattgctttgtgtgtgttacctgctctaagaagctggctgggcagcgtttcaccgctgtggaggaccagtattactgcgtggattgctacaagaactttgtggccaagaagtgtgctggatgcaagaaccccatcactgggaaaaggactgtgtcaagagtgagccacccagtctctaaagctaggaagcccccagtgtgccacgggaaacgcttgcctctcaccctgtttcccagcgccaacctccggggcaggcatccgggtggagagaggacttgtccctcgtgggtggtggttctttatagaaaaaatcgaagcttagcagctcctcgaggcccgggtttggtaaaggctccagtgtggtggcctatgaaggacaatcctggcacgactactgcttccactgcaaaaaatgctccgtga

The sequences of gRNAs were as follows:

gRNA-A1:GCCAGACCTTATGCTGGCTAAGG

gRNA-A2: CTCAGTCGGGACGCACTGTGCGG

gRNA-B1: GCACTACTGCGCATGTACCTAGG

gRNA-B2: GCCCACTCTTGCCCTATGGATGG

Primers for genotyping were designed and sequences:

Primers1:

F1:5’-TGGCAGGAAGGCTTCTGGCTT-3’

R1:5’-GCCACATAGGGGAGTCAAACATTA-3’; Product size: 488 bp

Primers2:

F1: 5’-TGGCAGGAAGGCTTCTGGCTT-3’

R2: 5’-CAGCTACAACAGTTACCTTAGCATC-3’; Product size: 778 bp

(Fhl1 KO product length=488 bp, wildtype product length=778 bp)
